# Single Disulfide Bond in Host Defense Thanatin Analog Peptides: Antimicrobial Activity, Atomic-Resolution Structures and Target Interactions

**DOI:** 10.3390/ijms26010051

**Published:** 2024-12-24

**Authors:** Swaleeha Jaan Abdullah, Jia Sheng Guan, Yuguang Mu, Surajit Bhattacharjya

**Affiliations:** School of Biological Sciences, Nanyang Technological University, Singapore 637551, Singapore

**Keywords:** host defense antimicrobial peptide, thanatin, NMR, LPS, LptA, LptA_m_

## Abstract

Host defense antimicrobial peptides (AMPs) are promising lead molecules with which to develop antibiotics against drug-resistant bacterial pathogens. Thanatin, an inducible antimicrobial peptide involved in the host defense of *Podisus maculiventris* insects, is gaining considerable attention in the generation of novel classes of antibiotics. Thanatin or thanatin-based analog peptides are extremely potent in killing bacterial pathogens in the Enterobacteriaceae family, including drug-resistant strains of *Escherichia coli* and *Klebsiella pneumoniae*. A single disulfide bond that covalently links two anti-parallel β-strands in thanatin could be pivotal to its selective antibacterial activity and mode of action. However, potential correlations of the disulfide covalent bond with structure, activity and target binding in thanatin peptides are currently unclear to. Here, we examined a 16-residue designed thanatin peptide, namely disulfide-bonded VF16QK, and its Cys to Ser substituted variant, VF16QK^Ser^, to delineate their structure–activity relationships. Bacterial growth inhibitory activity was only detected for the disulfide-bonded VF16QK peptide. Mechanistically, both peptides vastly differ in their bacterial cell permeabilizations, atomic-resolution structures, interactions with the LPS-outer membrane and target periplasmic protein LptA_m_ binding. In particular, analysis of the 3-D structures of the two peptides revealed an altered folded conformation for the VF16QK^Ser^ peptide that was correlated with diminished LPS-outer membrane permeabilization and target interactions. Analysis of docked complexes of LPS–thanatin peptides indicated potential structural requirements and conformational adaptation for antimicrobial activity. Collectively, these observations contrast with those for the disulfide-bonded β-hairpin antimicrobial protegrin and tachyplesin peptides, where disulfide bonds are dispensable for activity. We surmise that the atomistic structures and associated molecular interactions presented in this work can be utilized to design novel thanatin-based antibiotics.

## 1. Introduction

At present, the mitigation of infections arising from drug-resistant bacterial pathogens by the use of conventional antibiotics is complex and currently there are limited options to treat drug-resistant infections [1,2,3]. The global burden on human health due to antimicrobial resistance (AMR) is extremely concerning. A systematic analysis reported by the Antimicrobial Resistance Collaborators revealed an estimated five million deaths associated with bacterial AMR, of which 1.27 million were directly attributable deaths, in only the year of 2019 [4]. A similar study in the European region reported estimated fatalities of 541,000 and 133,000 people associated with and attributable to bacterial AMR, respectively [5]. Four Gram-negative bacteria (*Escherichia coli*, *Klebsiella pneumoniae*, *Acinetobacter baumannii* and *Pseudomonas aeruginosa*) and two Gram-positive bacteria, (*Staphylococcus aureus* and *Streptococcus pneumoniae*) have been identified as leading pathogens which are responsible for most AMR deaths [4,5]. Notably, a large number of bacterial AMR-attributable deaths in 2019 were caused by infections of *Escherichia coli* and *Klebsiella pneumoniae* belonging to the Enterobacteriaceae family [4,6]. As a matter of fact, nosocomial infections caused by drug resistant Gram-negative bacteria are extremely difficult to mitigate [7,8,9]. The LPS-outer membrane and efflux pumps are the significant challenges in the discovery of new antibiotics against drug- or multi-drug-resistant Gram-negative pathogens [10,11,12,13]. Many large scaffold frontline antibiotics, e.g., vancomycin, erythromycin, etc., are considered ineffective for the treatment of Gram-negative bacterial infections as they are unable to overcome the LPS-outer membrane permeability barrier [11,12,13]. Therefore, new antibiotics are urgently required to combat emerging pan-drug-resistant Gram-negative bacterial pathogens [14,15,16].

Antimicrobial peptides (AMPs) are naturally occurring host defense molecules which have high potential in designing antibiotics to treat infections caused by drug-resistant bacteria including Gram-negative strains [17,18,19,20]. As a mode of action, many cationic AMPs disrupt the bacterial plasma membrane following either the barrel stave, toroidal pore or carpet mechanisms [21,22,23]. Owing to their cationic properties, AMPs can permeabilize the LPS-outer membrane of Gram-negative bacteria, which is a pivotal step for gaining access to inner cell components including the plasma membrane [13,24,25,26,27]. The membrane-lytic mechanisms of AMPs are, however, non-selective and can be responsible for toxicity in clinical applications [28,29,30]. A class of AMPs, termed as outer membrane protein targeting antibiotics (OMPTAs), permeabilize the LPS-outer membrane and inhibit OM biogenesis [31,32]. These AMPs are extremely potent in killing MDR Gram-negative bacteria with high selectivity and low human toxicity [31,32]. Thanatins are a group of OMPTA β-hairpin AMPs which are expressed in several species of insects to defend against microbial infections [33,34,35,36]. The first thanatin peptide was isolated and characterized from the fat body of the spined soldier bug, *Podisus maculiventris*, induced upon bacterial challenge [33]. The 21-residue-long thanatin of *P. maculiventris* is well studied in the family in terms of activity, structure and mode of action [37,38,39]. Thanatin and its 16-residue shorter derivatives are promising peptide antibiotics against infections caused by drug-resistant strains of Enterobacteriaceae including *E. coli*, and *K. pneumoniae* [40,41,42,43]. All of the members of thanatins including *P. maculiventris* thanatin contain a conserved disulfide bond (i to i+7) to an eight-amino-acid cationic/polar loop [33,34,35,44]. Surrogating a covalent bond akin to the disulfide could be a strategy to design in vivo stable thanatin antibiotic peptides [45,46]. However, correlation of the single disulfide bond in the structure of thanatins with their antimicrobial activity and mode of action is yet to be firmly established [44,47,48]. In particular, a previous study reported that the disulfide bond of thanatin is dispensable for its antimicrobial activity and β-sheet conformation [47]. Other studies, however, have demonstrated the importance of the disulfide bond of thanatin in its antibacterial activity [44,48]. Here, we utilized the disulfide-bonded thanatin analog peptide VF16QK and its variant VF16QK^Ser^ where both the Cys residues were replaced with Ser. Our studies revealed that the removal of the single disulfide bond in the thanatin AMP VF16QK^Ser^ stabilizes non-native folded conformations lacking antibacterial activity. The VF16QK^Ser^ peptide showed impaired OM permeabilization and low affinity binding to the periplasmic protein LptA_m_. Structural and biophysical analyses of the thanatin AMPs presented here demonstrate disparate conformational adaptations and mechanisms of bacterial cell killing exist across disulfide-bonded AMPs.

## 2. Results

### 2.1. Thanatin Analog Peptides and Antibacterial Activity

Deletion of the five N-terminal residues of full-length thanatin do not affect its antimicrobial activity [40,42,43]. We designed the 16-residue thanatin antibacterial peptide VM16 (VPIIYCNRRTGKCQRF-amide) and the D-Lys-containing analog VF16 (VPIIYCNRRT-dk-KCQRF-amide) [42]. Notably, unlike most membrane-active AMPs, thanatin contains as many as three polar amino acids, N7, T9 and Q13, in its sequence. These residues can be replaced with cationic amino acids to improve membrane interactions. Here, we further modified VF16 (VPIIYCNRRT-dk-KCQRF-amide) to VF16QK (VPIIYCNRRT-dk-KCKRF-amide), whereby the polar Gln was substituted with cationic Lys to further enhance membrane disruption. To interrogate the role of the disulfide bond in activity and structure, two Cys residues were substituted with the isosteric residue Ser in the VF16QK^ser^ analog. The use of reducing agents to break disulfide bonds in peptide sample preparation may cause potential complications in activity assays and NMR analysis.

The antibacterial activity of the VF16QK and VF16QK^ser^ peptides against ATCC strains of *E. coli*, *K. pneumoniae*, *S. enterica*, *S. pyogenes* and *E. faecalis* were determined. As expected, VF16QK was highly potent in inhibiting the growth of Gram-negative and Gram-positive bacterial strains with MIC values ranging from 1 to 4 μM (Table 1), whereas the antibacterial activity of the VF16QK^ser^ peptide was found to be largely lacking, with MIC values > 16 μM (Table 1).

### 2.2. Outer Membrane Permeabilization and Surface Charge Neutralization

**Figure 1:**
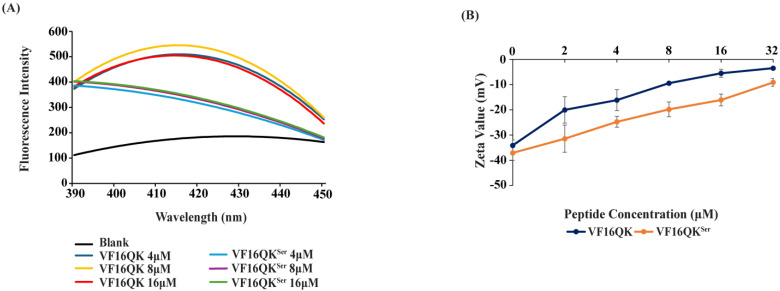
(**A**) Fluorescence emission spectra of NPN as a function of concentrations of the VF16QK and VF16QK^ser^ peptides in *E. coli* cell solutions. 10 μM of fluorescence probes were prepared in 10 mM of sodium phosphate buffer with a fixed bacterial cell density of 0.5. Fluorescence spectra were recorded with an excitation wavelength of 350 nm and emission of 390–450 nm. (**B**) Plot showing z potential changes of *E. coli* cells as a function of concentrations, 2 to 32 μM, of the VF16QK and VF16QK peptides.

The thanatin peptides VF16QK and VF16QK^ser^ were examined for their ability to permeabilize the LPS-outer membrane and interactions with surface charge and LPS. We assayed the fluorescence emission of NPN, a hydrophobic probe, upon additions of the VF16QK and VF16QK^ser^ peptides in *E. coli* cell solutions. NPN is excluded by the bacterial LPS-outer membrane, limiting fluorescence intensity. However, NPN can insert into the non-polar milieu of the LPS-outer membrane in a permeabilized bacterial cell wall, displaying higher fluorescence intensity [49]. Figure 1A shows NPN fluorescence with increasing concentrations of the VF16QK and VF16QK^ser^ peptides. Higher emission was apparent for VF16QK in comparison to VF16QK^ser^, suggesting the absence of the disulfide bond in the VF16QK^ser^ peptide significantly diminished LPS-outer membrane permeabilization. Next, we performed zeta potential measurements of *E. coli* cell solutions with the two peptides to delineate the neutralization of surface charge. Gram-negative bacteria possess a negative zeta potential due to the anionic phosphate and carboxylate groups of the LPS outer membrane [50,51]. Cationic peptides and proteins binding to Gram-negative bacteria cause a surface charge neutralization that is often linked with the membranolytic activity of AMPs [50,51]. Figure 1B shows changes of zeta potential values of *E. coli* cells with additions of the VF16QK and VF16QK^ser^ peptides. As seen, zeta potential values became less negative as concentrations of peptides were increased. However, the VF16QK^ser^ peptide was less effective compared to its disulfide-bonded counterpart in neutralizing the surface charge of bacterial cells.

### 2.3. Binding Interactions of VK16QK and VF16QK^ser^ with LptA_m_ and the LPS-Outer Membrane

**Figure 2:**
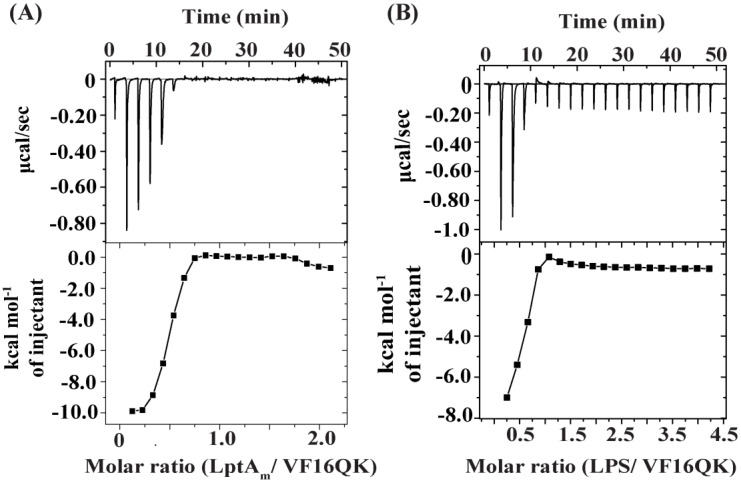
Isothermal titration calorimetry (ITC) of binding interactions of VF16QK with (**A**) LptA_m_, the periplasmic LPS transport protein of *E. coli*, and (**B**) the LPS-outer membrane. LptA_m_-peptide ITC studies were carried out in 50 mM of sodium phosphate buffer and 150 mM of NaCl at pH 7.0 and 25 °C. LptA_m_ proteins in sample cells were titrated with 2 μL aliquots of peptides and the heat exchange was measured. For LPS–peptide interactions, LPS in sample cells were titrated with 2 μL aliquots of peptides in 10 mM of sodium phosphate buffer, pH 7.0, 37 °C.

**Figure 3:**
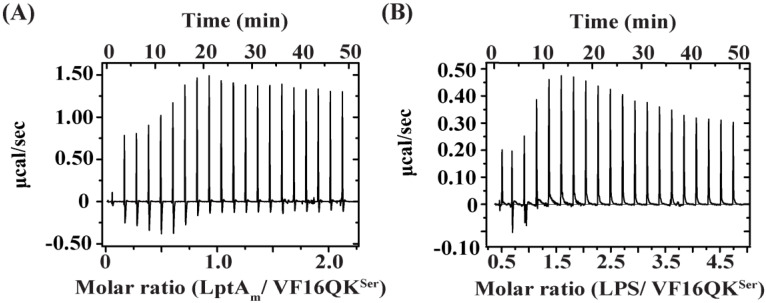
Isothermal titration calorimetry (ITC) of binding interactions of (**A**) LptA_m_ and (**B**) the LPS-outer membrane with the VF16QK^ser^ peptide.

Interactions of the peptides with the periplasmic protein LptA_m_ and LPS-outer membrane were determined using ITC experiments. Notably, LptA_m_ is a truncated but functional deletion variant of LptA and is known to bind thanatin peptides as a mode of bacterial cell killing [36,43,52]. LptA is a component of the complex of seven proteins (LptA-G) which is involved in transporting newly synthesized LPS from the inner membrane to the outer membrane [52,53]. In separate experiments, peptides were titrated into the sample cell containing either LptA_m_ or LPS and the heat of exchange was determined (see Section 4 Materials and Methods). LptA_m_ and LPS binding of the disulfide-bonded VF16QK peptide were found to be exothermic in nature (Figure 2A,B). The binding and thermodynamic parameters are listed in Table 2. The VF16QK peptide binds to LptA_m_ and the LPS-outer membrane with estimated K_d_ values of 0.4 μM and 2.11 μM, respectively (Table 2). Interestingly, by contrast, ITC revealed an endothermic binding of the VF16QK^ser^ peptide to LptA_m_ and LPS-outer membrane, which was observed to be rather unsaturable (Figure 3). The binding affinity and thermodynamic parameters could not be reliably determined potentially due to transient interactions of the VF16QK^ser^ peptide with LptA_m_ and the LPS-outer membrane.

### 2.4. NMRs of the VF16QK and VF16QK^ser^ Peptides in Free Solution and in Complex of LPS

**Figure 4:**
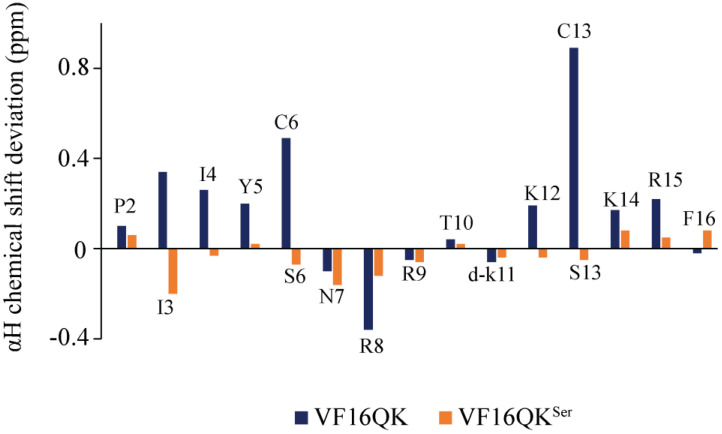
Bar diagram showing secondary chemical shifts of αH resonances of residues of the VF16QK and VF16QK^ser^ peptides.

**Figure 5:**
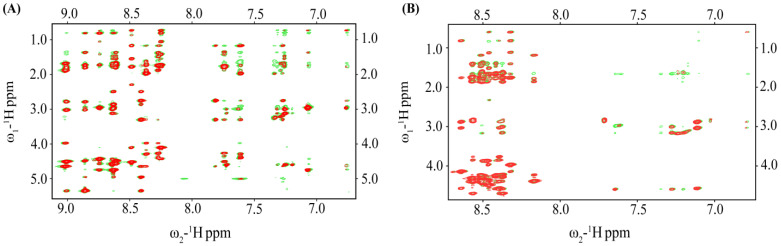
(**A**) Overlay of partial ^1^H-^1^H two-dimensional NOESY (red contour) and tr-NOESY (green contour) spectra of the VF16QK peptide and (**B**) overlay of partial ^1^H-^1^H two-dimensional NOESY (red contour) and tr-NOESY (green contour) spectra of the VF16QK^ser^ peptide. NOESY/tr-NOESY spectra show NOEs involving downfield shifted amide and aromatic proton resonances along the ω_2_ dimension with the upfield shifted aliphatic proton resonances along the ω_1_ dimension.

Sequence-specific resonance assignments of the VF16QK and VF16QK^ser^ peptides were obtained by two-dimensional TOCSY and NOESY. Figure 4 compares the secondary chemical shift of αH resonances of the two peptides. As seen, residues of the VF16QK^ser^ peptide delineated a marked diminution in secondary chemical shift values which are more pronounced for the β-strand residues, i.e., I3, I4, Y5, S6, K12 and S13, while the intervening residues, N7, R8, R9 and T10, at the loop/turn maintain the secondary chemical shift akin to disulfide-bonded VF16QK. Analyses of the NOESY spectra of VF16QK and VF16QK^ser^ demonstrated marked differences in long-range NOEs (Table 3 and Table 4). The disulfide-bonded VF16QK peptide delineated long-range NOE connectivities expected for a stable β-hairpin conformation (Table 3). A set of long-range NOEs could be detected for the VF16QK^ser^ peptide (Table 4), which were not observed for the parent VF16QK peptide. To determine structural transitions of the peptides in complex with the LPS-outer membrane, we carried out tr-NOESY experiments. New additional NOEs could be seen for the VF16QK peptide (Figure 5A). These NOEs are listed in Table 3. However, very few new NOEs were observed for the VF16QK^ser^ peptide (Figure 5B). Therefore, the aforementioned NMR analyses suggested that elimination of the single disulfide bond in the VF16QK^ser^ peptide appears to destabilize the canonical β-hairpin structure, whereas an altered conformation is likely to be stabilized. Furthermore, unlike the parent peptide, the interactions of the VF16QK^ser^ peptide with the LPS-outer membrane did not cause any structural changes.

### 2.5. Atomic-Resolution Structures of the VF16QK and VF16QK^ser^ Peptides

**Figure 6:**
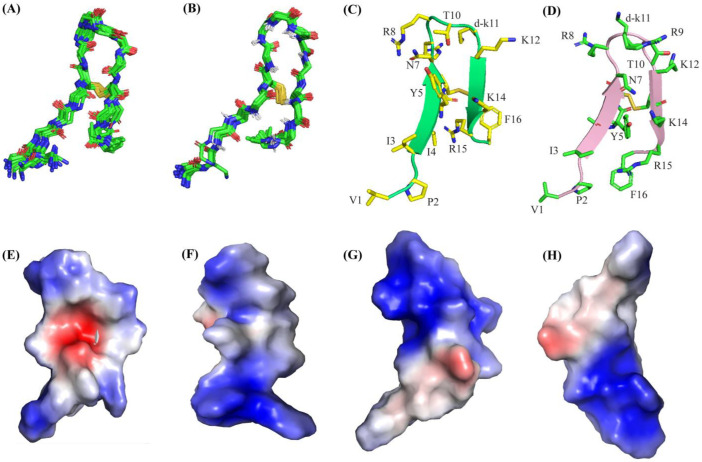
Superpositions of twenty CYANA-derived low-energy structures of the VF16QK peptide (**A**) in free solution (**B**) and in complex with LPS micelles. Ribbon representation showing backbone and sidechain orientations of the VF16QK peptide (**C**) in free solution and (**D**) in complex with LPS micelles. Electrostatic surface potential of the VF16QK peptide (**E**,**F**) in free solution and (**G**,**H**) in complex with LPS micelles.

Atomic-resolution structures of the VF16QK peptide were observed in free solution and in complex with the LPS-outer membrane using CYANA. Figure 6A,B shows backbone superpositions of twenty low-energy structures of the VF16QK peptide in free solution (Figure 6A) and in complex with LPS micelles (Figure 6B). As seen, structural ensembles of the peptide determined in the LPS-outer membrane are closely superposed compared to those of the free peptide. Table 5 summarizes the structural statistics derived for the twenty low-energy structures.

**Figure 7:**
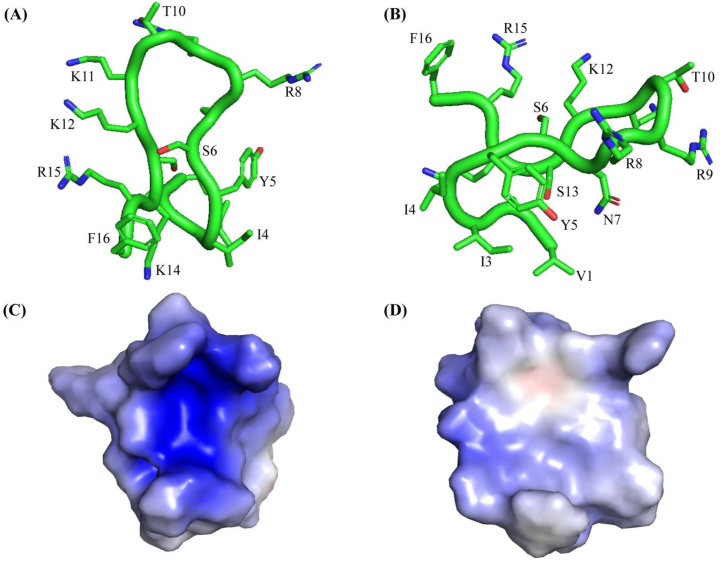
(**A**,**B**) Ribbon representation of the structure of the VF16QK^ser^ peptide in free solution showing backbone folding and sidechain dispositions in two different orientations. (**C**,**D**) Electrostatic surface potential of the structure of the VF16QK^ser^ peptide in free solution in two different orientations.

The VK16QK peptide assumed a β-harpin structure either in free solution (Figure 6C) or in complex with LPS (Figure 6D). The β-hairpin structure is defined by two anti-parallel β-strands, residues I2-I3-Y4-C5 and residues K12-C13-K14-R15 that are connected by a loop, residues N7-R8-R9-T10-K11 (Figure 6C,D). However, the sidechains, particularly cationic residues in the loop and in the β-strands, showed certain differences between the two structures (Figure 6D). The reorganization of the sidechains of cationic residues is emphasized by the well-defined electrostatic surface of the VF16QK peptide in complex the with LPS-outer membrane (Figure 6G,H), while the electrostatic surface of the structure in free solution appeared to be more scattered in the spatial position (Figure 6E,F). The NMR structure of the VF16QK^ser^ peptide was determined in free solution. Figure 7 shows the backbone topology and orientation of the sidechains of the structure of the VF16QK^ser^ peptide. The backbone folding delineates the anti-parallel positioning of the N- and C-terminal residues, although sidechain/sidechain interactions are limited within the structure (Figure 7A,B). Six cationic residues, R8, R9, K11, K12, K14 and R15, appear to form a distinct surface occupying the face of the structure of the VF16QK^ser^ peptide (Figure 7C,D).

### 2.6. Docked Structure of VK16QK and LPS

**Figure 8:**
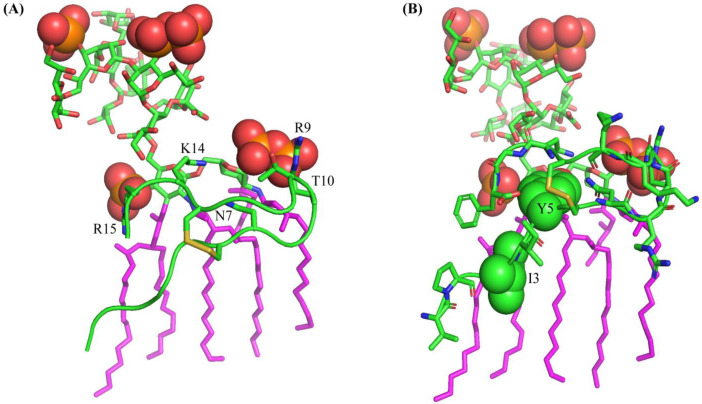
Docked structure of LPS-VF16QK showing (**A**) potential ionic and polar interactions among residues R9, N7, T10, K14 and R15 with the phosphate groups (in red sphere) of the lipid A moiety of LPS and (**B**) potential non-polar packing interactions of the residues I3 and Y5 with the acyl chain of LPS.

A docked structure of the complex shows several residues of the VK16QK peptide are in close proximity to the lipid A moiety of LPS (Figure 8). The cationic sidechains of residues R9 and R15 can form potential ionic and hydrogen bond interactions with the negatively charged phosphates of lipid A (Figure 8). The sidechain of residue K14 is also in proximity to the two phosphate groups of lipid A. In addition, potential packing or van der Waals’ interactions can be realized between the aromatic sidechain of residue Y5 and glucosamines of lipid A (Figure 8). Additional polar interactions are also probable involving the sidechains of residues N7 and T10 and the phosphates and glucosamines of lipid A. Moreover, non-polar packings are viable with the sidechain of residue I3 and acyl chain of lipid A.

## 3. Discussion

Disulfide bonds are known to occur in several AMPs, indicating potential linking between conformation and activity [54,55]. In particular, the β-sheet or β-hairpin AMPs are characterized by the presence of single or multiple disulfide bonds. The inter β-strand disulfide bonds impart stabilization to folded conformations in free solution and in membrane environments [56,57,58]. However, correlations of antimicrobial activity with disulfide bon-stabilized structures can be ambiguous. Studies have demonstrated that disulfide bonds in the β-hairpin protegrin and tachyplesin AMPs and β-sheet defensin antimicrobial protein are dispensable for their antimicrobial activity [59,60,61,62]. Disulfide-deleted analogs of tachyplesin and protegrin were found to possess antimicrobial activity and assumed β-hairpin conformations in complex with the LPS-outer membrane [63,64]. Previous studies have shown that deletion of the first six N-terminal amino acids of the 21-residue-long thanatin does not affect antimicrobial activity and bacterial target interactions [33,40,42,43]. However, the structural requirements of the single disulfide bond in thanatin and its active analogs are not well understood. A recent report demonstrated replacement of the disulfide bond with penicillamine could improve the in vivo stability and antibacterial activity of 16-residue analog peptides [40]. In this work, we examined structure–activity correlations of the disulfide bonds of the thanatin analog peptides VF16QK and VF16QK^ser^. Our studies disclosed that the disulfide bond perhaps is critical for the antibacterial activity of thanatin analog peptides. The disulfide-bonded VF16QK peptide exerted potent antibacterial activity whilst the Cys to Ser substituted variant VF16QK^ser^ was found to be largely inactive, although the disulfide sans peptide was able to permeabilize the LPS-outer membrane and neutralize the surface charge of bacterial cells. However, VK16QK displayed superior activity in cell wall permeabilization and charge neutralization. To further correlate, we determined binding interactions of the two peptides with the LPS-outer membrane and periplasmic LPS transport protein LptA_m_ and determined their atomic solution structures. ITC-derived interaction analyses showed that exothermic binding of the VF16QK peptide with LptA_m_ and the LPS-outer membrane was largely driven by favorable changes in enthalpy. Interestingly, the VK16QK^ser^ analog exhibited an endothermic binding interaction with LptA_m_ and the LPS-outer membrane. The binding of VK16QK^ser^ with LPS and LptA_m_ appeared to be non-saturable and binding parameters could not be estimated. Therefore, the two peptides VF16QK and VK16QK^ser^ demonstrated vast differences in their interaction characteristics with the bacterial targets. In other words, covalent stabilization imparted by the disulfide bond in thanatin analog peptides is required for their antimicrobial activity and high affinity interactions with the LPS-outer membrane and LptA_m_. Specifically, the disulfide bond per se can be replaced by other covalent bonds that can stabilize the juxtaposition of the two anti-parallel β-strands in thanatin peptides. While the current work was on-going, a recent study demonstrated the inclusion of a covalent vinyl sulfide bond substituting the labile disulfide bond restores the antimicrobial activity of thanatin peptides [65]. Regardless, the atomic-resolution structures of VF16QK and VF16QK^ser^ were determined in free solution and in complex with the LPS-outer membrane for the VF16QK peptide. VF16QK adopted a canonical β-hairpin structure in free solution and also in complex with LPS. However, more inter-sidechain packings were deduced for the β-hairpin structure in complex with the LPS-outer membrane. The atomic structure of VF16QK in LPS displayed an organized positively charge surface and higher amphipathicity compared to that of the structure in free solution. The consolidated cationic surface of VF16QK could be critical for the efficient surface charge neutralization and permeabilization of the LPS-outer membrane. The docked structure of the complex of VF16QK with LPS indicated potential peptide–LPS binding modes and interactions. The peptide–LPS complex is largely driven by ionic or salt bridge and polar interactions involving anionic phosphate groups of the lipid A and the sidechains of multiple cationic residues from the loop and C-terminal β-strand of the hairpin structure. Notably, the exothermic binding, observed in ITC, of VF16QK with the LPS-outer membrane is in agreement with the docked structure. In contrast, the atomic structure of the VF16QK^ser^ peptide displayed an overall loop-like conformation in free solution, although th conformations and activity of the VF16QK^ser^ peptide could be occurring due to the hydrophilic Ser residues opposed to the non-polar Cys residues. Nonetheless, the loop structure showed a cationic surface which can potentially exhibit transient interactions with the negatively charged LPS-outer membrane limiting membrane permeabilization and surface charge neutralization. Therefore, a stable β-hairpin structure is required for high affinity binding of the thanatin peptides with the LptA_m_ which is vital for LPS transport and outer membrane biogenesis. In conclusion, the results presented in the current work strongly demonstrate critical involvement of the covalent bond, as in disulfide bond, in the structure and activity of thanatin analog peptides. The diversity of disulfide-bonded AMPs and their correlation with structure and antibacterial activity are of high significance for the future development of therapeutics. We surmise that the current study could pave the way for generating novel AMPs based on thanatins.

## 4. Materials and Methods

### 4.1. Determination of Minimal Inhibitory Concentration (MIC)

Antibacterial activities of the VF16QK and VF16QK^Ser^ peptides (GL Biochem, Shanghai, China) were determined using the broth dilution method. Bacterial strains of *Escherichia coli* ATCC 25922 (EC); *Salmonella enterica* ATCC 14028 (SE); *Klebsiella pneumoniae* ATCC 13883 (KP); *Acinetobacter baumannii* ATCC BAA-1798 (AB) *Streptococcus pyogenes* ATCC 19615 (SP) and *Enterococcus faecalis* ATCC 29212 (EF) were used for this study.

Typically, overnight cultures of bacterial cells were grown to mid-log phase and diluted in 2X Mueller Hinton (MH) broth (Millipore-Sigma^TM^, Saint Louis, MO, USA) to a final OD_600_ of 0.002. In a 96-well plate, 100 μL of the diluted bacterial solutions and 100 μL of the VF16QK and VF16QK^Ser^ peptides in concentrations ranging from 0.5 μM to 8 μM were incubated at 37 °C for 18 h. MIC value was estimated where no bacterial growth was observed at OD_600_.

### 4.2. Outer Permeabilization Assay

VF16QK and VF16QK^Ser^ were assessed for their ability to permeabilize the LPS-outer membrane of *E. coli* cells by use of the fluorescence probe 1-N-phenylnapthylamine (NPN). Bacterial cells were prepared from a mid-log culture in 10 mM of sodium phosphate with an adjusted OD_600_ ~ 0.5. A basal fluorescence of 10 mM NPN in bacterial suspension, in absence of peptide, was measured followed by sequential additions of peptides from 1 to 16 µM. Fluorescence experiments were conducted in a Cary Eclipse spectrophotometer (Varian, Palo Alto, CA, USA) using a 0.1 cm quartz cuvette. An excitation wavelength of 350 nm and emission was recorded from 390–450 nm.

### 4.3. Zeta Potential Measurements

Zeta potential measurements were performed in a Zeta Sizer Nano ZS (Malvern Instruments, Worcestershire, UK) instrument equipped with a 633 nm He laser. Mid-log phase *E. coli* cultures were centrifuged at 5000 rpm for 10 min and prepared in 10 mM of sodium phosphate buffer, pH 7. Z potential values of bacterial cell suspension were obtained from 1 to 32 μM of the peptides in zeta cells with gold electrodes. For individual measurement, 100 runs were carried out and three replicates were performed.

### 4.4. Isothermal Titration Calorimetry (ITC) Studies

The binding interactions of the thanatin peptides VF16QK and VF16QK^Ser^ with the LPS-outer membrane and LptA_m_ were determined using ITC. LPS and peptide samples were prepared in 10 mM of sodium phosphate buffer, pH 7.0. Typically, twenty injections, or 2.0 μL of peptides (1 mM in syringe), were titrated into the sample cell containing LPS (50 mM) at 37 °C with a stirring speed of 750 rpm. ITC titrations were performed with 20 injections of 2 µL of individual peptide (250 μM in syringe) into the sample cell containing LptA_m_ (25 mM) at 25 °C and stirred with a speed of 750 rpm. ITC data were analyzed following single site binding in Microcal origin 5.0 software to determine the association constant (K_a_) and change of enthalpy (ΔH). The dissociation constant (K_d_), change of free energy (ΔG) and change of entropy (TΔS) were calculated as: K_d_ = 1/K_a_, ΔG = ΔH − TΔS, respectively.

### 4.5. Purification of LptA_m_

His-tagged LptA_m_ (residues 28–159) of *E. coli* was recombinantly expressed in the *E. coli* BL21 cell line [66]. Bacterial competent cells were transformed by plasmid DNA coding for the LptA_m_ gene and spread onto LB agar plates with antibiotic selection, 100 μg/mL of ampicillin. Mid-log bacteria cell cultures, OD_600_~0.6, were induced with 1 mM isopropyl β-d-1-thiogalactopyranoside (IPTG), and were kept at 18 °C at 180 rpm for 18 h. Cells were centrifuged at 6000 rpm at 4 °C for 15 min and the pellet was resuspended in a buffer (100 mM of HEPES, 500 mM of NaCl, 10 mM of imidazole, pH 8). Cells were lyzed by sonication at 25 Amp for 1 h on ice and centrifuged at 18,000 rpm at 4 °C for 30 min. The cell supernatant was passed through Ni-NTA (Qiagen, Hilden, Germany) beads thrice before washing with increasing concentrations of imidazole. Bound His-tagged LptA_m_ was eluted from the beads with high imidazole buffer (20 mM of HEPES, 150 of mM NaCl, 200 mM of imidazole, pH 8). Protein was concentrated and further purified by size-exclusion chromatography using buffer (50 mM of sodium phosphate buffer, 150 mM of NaCl, pH 7).

### 4.6. NMR Experiments of the VF16QK and VF16QK^Ser^ Peptides

NMR studies were carried out using a Bruker DRX 600 spectrometer, equipped with a cryo-probe and pulse field gradients. NMR data were processed using TopSpin software 4.4.1 and analyzed by SPARKY (T.D. Goddard and D.G. Kneller, University of California, San Francisco, CA, USA). Peptide samples, 300 mM, were prepared in 10 mM of sodium phosphate buffer, pH 5.8, for acquiring two-dimensional TOCSY (total correlation spectroscopy) and NOESY (nuclear Overhauser effect spectroscopy) data at 278 K with mixing times of 80 ms and 300 ms, respectively. To determine the LPS-bound structures of peptides, 2-D transferred NOESY (tr-NOESY) experiments were performed in the presence of 20 μM LPS with a mixing time of 150 ms. Chemical shift values of resonances were referenced to DSS (2,2-dimethyl-2-silapentane 5-sulfonate sodium salt) at 0 ppm.

### 4.7. Structure Calculations for Peptides and Docking with LPS

The three-dimensional structures of the VF16QK and VF16QK^Ser^ peptides were determined using the CYANA program [67]. Distance constraints were obtained from either NOESY or tr-NOESY spectra, whereby cross-peaks were qualitatively classified as strong, medium and weak NOEs with upper-bound distance limits of 2.5, 3.5 and 5.0 Å, respectively, while the lower distant limit was capped at 2.0 Å. Additional constraints were included to form the disulfide bond between two cysteine residues for VF16QK. Angular (ϕ and φ) constraints were obtained from PREDITOR [68]. Of the total of 100 structures calculated, 20 structures with the lowest energy were selected to represent the ensemble and used for further analysis. The quality of the structures was assessed using PROCHECK107 [69]. LPS and VF16QK were docked using QuickVina2.

## Figures and Tables

**Table 1:** Antimicrobial activity of VF16QK and VF16QK^Ser^. Minimum inhibitory concentration (in µM) of VF16QK and VF16QK^Ser^ against the Gram-negative bacteria *Escherichia coli* ATCC 25922 (EC), *Salmonella enterica* ATCC 14028 (SE) and *Klebsiella pneumoniae* ATCC 13883 (KP) and the Gram-positive bacteria *Streptococcus pyogenes* ATCC 19615 (SP) and *Enterococcus faecalis* ATCC 29212 (EF).

VM16 Analogs	MIC (in µM)				
	Gram-Negative			Gram-Positive	
	EC	KP	SE	SP	EF
**VF16QK**	1–4	1–2	1–2	1	1
**VF16QK^Ser^**	>16	>16	>16	>16	>16

**Table 2:** Thermodynamic parameters of interactions of VF16QK with LptA_m_ and LPS.

	K_d_ (μM)	ΔH (kcal/mol)	TΔS (kcal/mol)	ΔG (kcal/mol)
**VF16QK-LptA_m_**	0.40	−10.16	−1.43	−8.73
**VF16QK-LPS**	2.11	−8.10	−0.05	−8.15

**Table 3:** Representative long-range NOEs of VF16QK in free solution and in complex of LPS.

Free VF16QK	VF16QK with LPS
I4 ^δ^H_3_–K14 H Y5 H–K14 H C6 ^α^H–K14 H N7 ^β^H_2_–K12 H K12 H–N7 H C13 ^α^H–N7 H K14 H–Y5 H K14 H–Y5 ^δ^H_s_ R15 ^α^H–Y5 H	I3 ^δ^H_3_–R15 ^Ɛ^H_s_ I4 ^δ^H_3_–K14 H Y5 H–K14 H C6 ^α^H–K14 H N7 ^β^H_2_–K12 H N7 H–K14 H K12 H–N7 H C13 ^α^H–N7 H K14 H–Y5 H K14 ^β^H_S–_Y5 ^δ^H_s_ K14 ^β^H_S–_Y5 ^δ^H_s_ K14 ^β^H_S_– 5 Y5 ^Ɛ^H_s_ K14 ^γ^H_S_–5 Y5 ^δ^H_s_ K14 H–C6 H R15 ^β^H_3_–Y5 ^δ^H_s_ F16^α^H–Y5H F16 H–Y5 H

**Table 4:** List of long-range NOEs observed for VF16QK^Ser^ in free solution.

VF16QK^Ser^
P2 ^α^H–K14 H S6 ^β^H_2_–K14 H S6 ^β^H_3–_K14 H S13 H–S6 H S13 ^α^H–N7 H K14 H–Y5 H

**Table 5:** Summary of structural statistics of VF16QK and VF16QK^Ser^ in both free solution and in LPS.

	Free VF16QK	VF16QK with LPS	Free VF16QK^Ser^
**Distance constraints**			
Intra residue [|i − j| = 0]	86	89	75
Sequential [|i − j| = 1]	46	55	23
Medium Range [1 < |i − j| < 4]	8	14	5
Long Range [|i − j| ≥ 4]	9	17	6
Total NOE	149	175	109
**Dihedral—angle constraints**	24	24	24
**Deviation from mean structure**			
All backbone atoms **(Å)**	0.90	0.44	1.36
All heavy atoms **(Å)**	1.62	1.15	2.54
**Ramachandran plot for the mean structure**			
% of residues in most favored region & Additional allowed region	100	100	100
% of residues in generously allowed region	0	0	0
% of residues in disallowed region	0	0	0

## Data Availability

Data is contained within the article.
